# Stereotactic Body Radiation Therapy in Gynecologic Oligometastases: An Effective but Underutilized Approach

**DOI:** 10.3390/cancers15133526

**Published:** 2023-07-07

**Authors:** Zohaib Sherwani, Shreel Parikh, Nikhil Yegya-Raman, Kelly McKenna, Matthew Deek, Salma Jabbour, Lara Hathout

**Affiliations:** 1 Department of Radiation Oncology, Rutgers Cancer Institute of New Jersey, Rutgers University, New Brunswick, NJ 08901, USAjabbousk@cinj.rutgers.edu (S.J.); 2 Department of Radiation Oncology, University of Pennsylvania, Philadelphia, PA 19104, USA

**Keywords:** stereotactic body radiation therapy, oligometastases, gynecologic cancer, ovarian cancer, cervical cancer, uterine cancer

## Abstract

**Simple Summary:**

Stereotactic body radiation therapy (SBRT) allows for the high-precision delivery of a large dose of radiation to a small area. SBRT has shown overall survival benefits in a variety of oligometastatic cancers in recent trials. However, prospective data in recurrent or oligometastatic gynecologic cancer are limited. The current literature shows that SBRT in gynecologic oligometastases is safe and effective, resulting in 2-year local control rates exceeding 80%. However, progression outside of the radiated field remains a significant issue despite local control. This review discusses the advantages, limitations, and future directions of SBRT in gynecologic oligometastases including a discussion on the synergistic effects of combination with immunotherapy.

**Abstract:**

Historically, the role of radiation in gynecological metastatic disease involved palliation for pain or bleeding. Stereotactic Body Radiation Therapy (SBRT) has shown survival benefits in oligometastatic disease from varying primary histologies in recent randomized trials. However, gynecologic primary oligometastases have been underrepresented in these trials. Recent studies across gynecological malignancy types have similarly shown favorable outcomes and acceptable toxicities from treating recurrent or oligometastatic gynecologic cancer (ROMGC) patients with definitive radiation therapy. The largest body of literature reported on the use of SBRT in ovarian cancer, which was found to be an effective option, especially in the setting of chemo-resistant disease. Despite the encouraging outcomes using SBRT in oligometastatic gynecologic malignancies, SBRT remains underutilized given the lack of randomized studies studying ROMGC with long term follow-up. While waiting for future prospective trials to establish the role of SBRT as the standard of care in ROMGC patients, this review focuses on reporting the advantages and drawbacks of this technique and examines the current literature to help guide patient centered treatment decisions.

## 1. Introduction

Stereotactic body radiation therapy (SBRT) is a promising technique in the local treatment of oligometastatic disease with the delivery of high biologically equivalent doses (BED) to small volumes of gross disease over a hypofractionated or abbreviated schedule. While there is no universally accepted definition for the oligometastatic state, it commonly refers to an intermediate state between locally advanced and widely metastatic disease, defined as the presence of 1–5 metastases in which radical local treatment might improve systemic control [1,2,3,4]. The maximum number of metastases that can be treated is not agreed upon. In a consensus guideline recently published by ESTRO-ASTRO [5], “the feasibility of safely delivering curative intent metastasis-directed radiation therapy determines the maximum number of lesions and sites”. As a result, SBRT-directed local therapy to oligometastatic sites offers a potential for systemic control; and the number of lesions treated are largely dependent on the safe administration of large doses of radiation.

The SABR-COMET phase II clinical trial randomized 99 patients with oligometastatic cancer and multiple tumor types (lung, breast, prostate, etc.) in a 1:2 ratio to palliative standard of care treatment versus Stereotactic Ablative Radiotherapy (SABR) to all metastases plus standard of care. Patients must have had a controlled primary tumor and 1 to 5 metastases amenable to SABR. SABR improved median overall survival from 28 months to 41 months and exhibited an overall favorable toxicity profile [1]. However, only 2 out of the 99 total patients were reported to have a gynecologic primary. Several studies were published over the last decade evaluating the role of SBRT in recurrent or oligometastatic gynecologic malignancies. To our knowledge, no prospective randomized study specifically evaluated the impact of SBRT in recurrent or oligometastatic gynecologic cancer (ROMGC). Most studies were retrospective, single institutional, and did not differentiate between primary malignancies. This review focuses on reporting the role of SBRT in ROMGC, including the advantages and drawbacks from this technique, by examining the current literature to guide radiation oncologists in patient-centered decision making.

## 2. SBRT in Ovarian Primary Oligometastases

Radiation was historically used to treat metastatic ovarian cancer in a palliative setting, with a well-established efficacy in the treatment of pain and bleeding [6,7,8]. Local recurrence within the abdomen or pelvis remained a significant factor contributing to poor prognosis despite multiple courses of chemotherapy. Definitive intent treatment using involved field radiation therapy (IFRT) resulted in modest benefits in overall survival and in-field disease control [9,10]. Intensity-modulated radiation therapy (IMRT) was more promising, with 2-year local control (LC) and overall survival (OS) rates of 82% and 63%, respectively, in refractory disease despite a median of three regimens of chemotherapy for recurrence within the abdomen or pelvis [11]. More recently, the use of SBRT has been evaluated in oligometastatic ovarian cancer.

A recent systematic review on SBRT in gynecologic malignancies [12] analyzed sixteen studies including one prospective phase II trial, two prospective phase I trials and thirteen retrospective reviews dating back to 2009. A total of 667 patients with 1071 oligometastatic lesions from varying ROMGC histological subtypes were evaluated. More than half (57.6%) of patients had primary ovarian oligometastases and 65.4% had a single lesion treated. Most lesions that were treated were at nodal sites (64%) within the abdomen (44.2%) or pelvis (18.8%) to a median BED_10_ of 50.7 Gy with response rates and local control exceeding 75% and 80%, respectively. Despite the high local control rates, disease progression occurred outside of the SBRT field. In addition, over half (56%) of the studies found no grade 3 or higher toxicities, suggesting that SBRT is well tolerated overall. Although this is representative of a mixed cohort of primary malignancies, a significant portion of patients in this review are from one retrospective review, the Multicenter Italian Trial in Ovarian cancer and gynecologic (MITO) RT1 study, which specifically evaluated the role of SBRT in ovarian primary oligometastases.

The MITO RT1 study is the largest retrospective review of ovarian primary oligometastases treated with SBRT, including 261 patients with 449 oligometastatic lesions with a median follow up of 22 months [13]. Most patients had a single lesion (55.9%) in abdominal lymph nodes (65%) with a median size of 15.7 cc. Inclusion criteria included any site of disease and up to five synchronous lesions. The most common histology was high-grade serous in 71.3% of patients. All patients underwent chemotherapy prior to radiation and 88.2% were treated with SBRT while 11.8% were treated with single-fraction radiotherapy (SRS). The median SBRT dose was 27 Gy (range 18–75) with a median BED_10_ of 48 Gy (range, 28–262.5). The most frequent SBRT prescriptions were 8 Gy × 3 fractions, 5 Gy × 5 fractions and 9 Gy × 3 fractions. Complete response was achieved in 65.2% of patients, with a 2-year actuarial local control of 81.9%, 2-year progression-free survival of 15.4%, and 2-year overall survival of 73.6%. On multivariate analysis, the following factors were identified as independent predictors for a higher likelihood of complete response and increased local control: patients younger than 60 years old, planning target volume (PTV) < 18 cc, lymph node disease and BED_10_ > 70. SBRT was well tolerated with 95.1% late toxicity-free survival.

Two other studies have reported comparable complete response rate with SBRT in oligometastatic ovarian cancer. An Italian retrospective study reported their experience with SBRT in 26 patients with 44 metastatic lesions [14]. Most patients had lymph node metastases (63.6%) treated to a median total dose of 45 Gy (range, 36–60 Gy) in six fractions (range, 4–8). After a median follow-up of 28.5 months, complete response was 59.1%, the 2-year local control was 92.9% and the median progression-free survival was 19 months. No grade 3 or 4 toxicities were reported [14].

Similarly, a single-institution retrospective study by Lazzari et al. evaluated the efficacy of SBRT in patients with oligometastatic ovarian cancer ineligible for surgery or systemic therapy [15]. A total of 82 patients with 156 lesions were included and received a median dose of 24 Gy in three fractions. Complete response was reported in 60% of patients after a median follow-up of 17.4 months. The median systemic treatment-free interval after SBRT was 7.4 months while the 2-year local progression-free survival was 68%. Most failures (90%) were outside of the radiation field [15].

Overall, SBRT is an effective and well-tolerated treatment option for ovarian primary oligometastases, with high rates of complete response and local control, as well as favorable overall survival and low rates of late toxicity. Prospective studies are ongoing, including the MITO RT3/RAD study (NCT04593381) [16], which is a multicenter prospective phase II study evaluating SBRT for the treatment of oligometastatic, persistent or recurrent ovarian cancer not amenable to surgery, local therapy and systemic therapy. SBRT is delivered in one, three or five daily fractions to a total dose of 30–50 Gy to all sites of active metastatic disease reported on imaging. The primary endpoint is a clinical complete response on a per lesion basis which aims to complete enrollment and analysis in 2023.

While awaiting the results of prospective data, select patients with oligometastatic, recurrent or persistent ovarian cancer should be considered for treatment with SBRT especially in patients who are not candidates for surgery and systemic therapy.

## 3. SBRT in Non-Ovarian Gynecologic Primary Oligometastases

Most retrospective studies evaluating the role of SBRT in gynecologic malignancies included multiple primary histologies; although ovarian cancer was studied most, cervical, and uterine cancer were included as well [Table 1]. There are no trials to our knowledge with significant numbers of patients with vaginal or vulvar primary oligometastases or intracranial metastases, and thus will not be a focus of this review. Cuccia et al. reported the outcomes of 40 patients with 60 extracranial oligometastases from gynecological cancers. Metastases were from ovarian cancer (43%), endometrial cancer (41%), cervical cancer (13%) and vaginal cancer (3%). Lymph node metastases were the most common site of radiation (55.5%) and most patients had a single lesion (65%). SBRT was delivered to a total median dose of 42 Gy (range, 24–70) and a median BED_10_ of 72 Gy_10_ (range, 48–180 Gy_10_). After a median follow-up 27 months, the median local control was 19 months, the 2-year local control rate was 100% and the 2-year progression-free survival was 23%.

In a retrospective study by Onal et al. [17], 29 patients with 35 oligometastatic cervical cancer (72%) and ovarian cancer (28%) were treated with SBRT for either de novo oligometastatic disease (24%) or oligoprogressive disease (76%). The 2-year local control was 85% and disease progression occurred at a median time of 7.7 months after SBRT. Complete response after SBRT was correlated with improved overall and progression-free survival.

**Table 1:** Recent studies in recurrent or oligometastatic gynecologic cancers treated with SBRT.

Year	Authors	Type of Study	# Patients (n)	# Lesions (n)	MFU (Months)	Outcomes	Toxicity	Progression
2018	Iftode et al. [14]	R	Ovarian n = 26	44	28.5	2yr LC 92.9% 2yr PFS 38% 2yr OS 92.7%	G2 = 11.3% No ≥ G3	7.7% L 3.8% LD 50% D
2020	Kowalchuk et al. [18]	R	Ovarian n = 35	98	33.67	2yr LC 80% 2yr PFS 12% 2yr OS 60%	27 cases < G3 Single G5 duodenal ulcer	17% L 32% LD 39% D
2018	Lazzari et al. [15]	R	Ovarian n = 82	156	17.4	2yr Local PFS 68% 2yr PFS 18% 2yr OS 71%	Acute G1 − G2 = 27% Late G1 − G2 = 28% No ≥ G3	3.5% L 5.5% LD 90% D
2020	Macchia et al. [13]	R	Ovarian n = 261	449	22	2yr LC 81.9% 2yr PFS 15.4% 2yr OS 73.6%	Acute G1 − G2 = 20.7% Late G1 − G2 = 6.1% 2yr late toxicity free survival = 95.1%.	18.1% L 84.6% LD/D
2022	Macchia et al. [19]	R	Cervical n = 83	125	14.5	2yr LC 61.8% 2yr PFS 28.9% 2yr OS 59%	Acute G1 − G2 = 18.1% Late G1− G3 = 4.8% Single G3 pain toxicity	38.2% L 71.1% LD/D
2020	Reddy et al. [20]	R	Uterine n = 27	61	16.9	1yr Local PFS 75.9% 1yr OS 65.4%	Total G1 − G2 = 29.6% of which acute = 93.3% & Late = 6.6% No ≥ G3	7.4% L 74.1% LD
2020	Onal et al. [17]	R	Ovarian n = 21 Cervical n = 8 Total = 29	35	15.3	2yr LC 84% 2yr PFS 18% 2yr OS 62%	G2 = 17% No ≥ G3	11% L 84% D
2020	Aghdam et al. [21]	R	Ovarian n = 10 Uterine n = 10 Total = 20	20	56	5yr LC 73% 5yr PFS 20% 5yr OS 56%	Single G3 MSK toxicity	NR
2020	Reshko et al. [20]	R	Ovarian n = 30 Cervical n = 20 Uterine n = 27 Vaginal n = 8 Vulva = 1 Total = 86	209	20	1yr LC 80% 1yr OS 70%	4.3% ≥ G2 Single G3 GU toxicity	NR
2020	Yegya-Raman et al. [12]	SR	Ovarian n = 384 Cervical n = 181 Uterine n = 74 Vaginal n = 3 Vulvar n =2 Other/NS = 23 Total = 667	1071	22 (range 4.6-54.6)	2yr LC 71–100% 2yr PFS 15.4–48.4% 2yr OS 57.5–85%	No ≥ G3 toxicity in 9/16 studies	Progression in 23.1–75% patients. 78.9–100% of progression including out-of-field component
2021	Cuccia et al. [22]	R	Ovarian n = 17 Cervical n = 4 Uterine n = 17 Vagina n = 2 Total = 40	63	27	2yr LC 100% 2yr PFS 23% 2yr OS 70%	No acute or late ≥ G2 toxicity	NR

R = retrospective, SR = systematic review, MFU = median follow up, LC = local control, PFS = progression free survival, OS = overall survival, L = local (in -field) progression, LD = Local/distant (in & out-of-field) progression, D = distant (out-of-field) progression, NR = not reported, NS = not specified.

SBRT has been evaluated in primary oligometastatic cervical cancer in the recently published MITO RT2/RAD study [19] which represents the largest retrospective cohort of patients with oligometastases from primary cervical cancer treated with SBRT. A total of 83 patients with 125 oligometastatic cervical cancer lesions were treated with SBRT. Most patients had single (69.9%) pelvic (36.8%) lymph node (55.2%) disease treated to a total dose of 35 Gy in five fractions with a median BED_10_ of 59.5 Gy. After a median follow-up of 14.5 months, complete response was noted in 58.4% of lesions and in 55.4% of patients. Interestingly, the authors noted significant improvements in local control, progression-free survival and overall survival in patients who had complete response (CR), as opposed to any other response (partial response (PR), stable disease (SD) or progression of disease (PD)). Two-year local control was 89% for CR and 22.1% for PR/SD/PD, respectively. Two-year progression-free survival was 42.5% for CR and 7.8% for PR/SD/PD, respectively. Two-year overall survival was 68.9% for CR and 44.4% for PR/SD/PD, respectively. However, no factors independently predicted for complete response. 

While the MITO RT1 study demonstrated the effectiveness and tolerability of SBRT for ovarian primary oligometastases, the MITO RT2/RAD study showed less favorable outcomes in cervical primary oligometastases treated with SBRT, but still suggested a promising avenue for further research in patients with comparatively unfavorable prognostic factors. In fact, early evidence in patients with cervical cancer with supraclavicular lymph node involvement without distant metastases showed that treatment with standard fractionation led to long term disease control which helped provide early rationale for selective targeting of oligometastatic disease with curative intent [23,24,25].

As for uterine cancer, most of the current data regarding uterine primary oligometastases are from retrospective studies with mixed cohorts [21,22,26,27,28,29,30]. The only recent study to our knowledge that exclusively included uterine primary oligometastases treated with SBRT is a single-institution retrospective analysis including 27 patients with 61 biopsy-proven lesions [20]. Most patients (74%) were treated for oligorecurrence and half (51.9%) had adenocarcinoma. The authors reported favorable response in 80.3% of cases and a median 1-year local progression-free survival rate of 75.9%, which was maintained at 3 years. The 1-year and 3-year overall survival rates were 65.4% and 28.7%, respectively. Liver lesions were found to be associated with a less favorable response on multivariate analysis, with only 37.5% of liver lesions with a favorable response. Tumor size < 3.8 cm was associated with a favorable response in the univariate analysis, which trended towards significance in the multivariate analysis.

**Figure 1:**
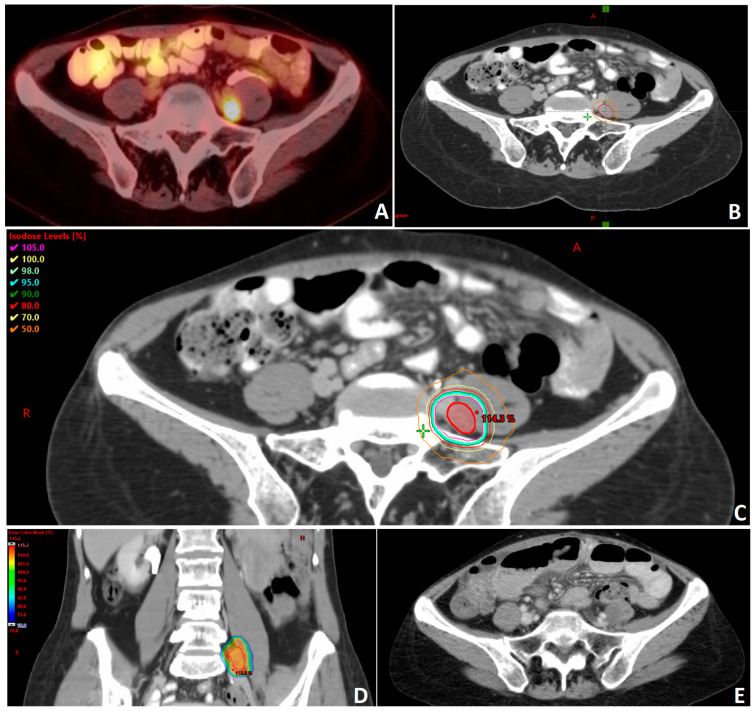
Clinical example of a patient with recurrent and oligometastatic high grade serous carcinoma of the ovary following surgery and multiple lines of systemic therapy. (**A**) Patient was found to have a hypermetabolic nodule medial to the left psoas muscle on positron emission tomography (PET) compatible with metastatic disease. (**B**) Gross tumor volume (GTV) delineation of oligometastatic nodule (red) with 0.5 mm planning tumor volume (PTV) radial expansion (orange). The treatment plan for this patient was generated to deliver a dose of 3000 cGy in 5 fractions prescribed to the PTV. The technique used in this plan is stereotactic body radiotherapy (SBRT) utilizing volumetric modulated arc therapy (VMAT) with a 10 MV beam energy. The plan is prescribed to the PTV. Axial (**C**) and coronal (**D**) images showing dose distribution. There is a max dose of 115.2% of the prescription dose with the PTV. (**E**) Representative axial image of follow-up computed tomography (CT) showing resolution of the treated nodule.

The delivery of a high BED over a small volume with rapid dose fall off is an advantage of SBRT, as seen in Figure 1. However, a limitation of SBRT is its inability to target the adjacent, potentially clinically relevant, at-risk nodal echelon. It has been commonly reported in the literature that despite local ablative radiation to oligometastatic sites, disease progression varies widely, with rates ranging between 23.1% and 75%. Disease progression outside of the radiated field remains the main site of progression and occurs in 78.9–100% of cases [12]. Superior overall survival and progression-free survival have been noted in early detection of oligometastatic lesions and complete response following SBRT [12,17,31], which may suggest micrometastatic spread in the neighboring lymphatics that is not clinically apparent at the time of evaluation, resulting in out-of-field progression despite local ablative radiation.

Given the high rate of disease progression outside of the SBRT field, elective nodal radiotherapy as metastasis-directed treatment was evaluated in a recently published landmark analysis [26], with a median follow up of 11.7 years. Definitive standard fractionation 2D/3D conformal radiation therapy (CRT) or IMRT including the entire at-risk nodal echelon was delivered to 48 patients with gynecologic oligometastases, resulting in disease-free survival and overall survival of 73 months and 200 months, respectively. Nodal sites were treated to a median dose of 62 Gy to the gross tumor volume (GTV) in 2.0–2.2 Gy per fraction with the clinical target volume (CTV) covering the adjacent nodal echelon to a dose of 45–50 Gy in 1.8–2.0 Gy fractions. Fifty percent of patients in this cohort had recurrence after a median of 28 months, predominantly with an out-of-field component, despite the extension of coverage to neighboring lymphatics. Interestingly, there was a significant improvement in disease-free survival in patients receiving any chemotherapy with radiation as opposed to radiation alone (93 months vs. 34 months, respectively). This highlights the importance of systemic therapies in the treatment of oligometastatic disease.

## 4. Radiation and the Impact of the Tumor Microenvironment

Understanding the complex interplay between the tumor microenvironment (TME), tumor stroma ratio (TSR), tissue hypoxia, and the effects of radiation therapy on cancer biology is essential for developing effective treatment strategies. The TME and TSR play critical roles in tumor progression, metastasis, and response to therapies, including radiation and immunotherapy. TME is a complex milieu composed of a stroma of various cell types, extracellular matrix (ECM) components, and signaling molecules that can promote tumor growth, angiogenesis, and immune suppression [32]. The TSR reflects the proportion of stromal tissue to cancerous tissue within a tumor, with a lower TSR indicating a more abundant tumor stroma. A low TSR, defined as greater than 50% of stroma on hematoxylin and eosin stains of surgical specimens, has shown a significant correlation with worse survival and more advanced disease. A low TSR was also correlated with worse clinicopathologic features in a wide variety of solid tumors including endometrial cancer in a systematic review and meta-analysis, although this effect was not seen in early cervical cancer [33].

Hypoxia, a common feature of the TME, contributes to tumor progression, therapy resistance, and immune modulation [34]. Hypoxia-driven angiogenesis results in abnormal, leaky blood vessels, further perpetuating a pro-tumorigenic microenvironment. Hypoxia can influence radiation response by reducing the effectiveness of ionizing radiation, as the latter relies on the presence of oxygen to create cytotoxic reactive oxygen species (ROS) that damage DNA [35,36,37]. In the case of SBRT, high doses of radiation can lead to more significant endothelial cell damage, potentially exacerbating hypoxia and limiting the efficacy of the treatment [38]. However, hypoxia-targeting strategies, such as combining radiation therapy with anti-angiogenic agents, can help overcome these challenges and enhance the therapeutic outcome [39].

SBRT, with the delivery of higher doses of radiation per fraction compared to standard fractionation, can lead to more pronounced immune-stimulatory effects. Hypofractionated regimens have been shown to modulate the tumor vasculature, potentially overcoming the negative effects of tissue hypoxia on radiation response. SBRT can induce a strong abscopal effect, wherein local radiation therapy results in the regression of distant, non-irradiated tumor sites through systemic immune activation [40,41,42,43,44].

While SBRT has shown favorable local control in ROMGC, out-of-field progression remains a significant cause of decreased overall survival. Additionally, radiation-induced damage in SBRT has been shown to trigger the immune response via the release of tumor antigen and damage-associated molecular patterns (DAMPs), causing the abscopal effect [45,46]. Although this phenomenon has been cited in the literature for decades, it has been only seen in case reports, pre-clinical and translational studies. The interactions between SBRT and checkpoint inhibitors have shown synergistic effects, resulting in improvements in overall survival and objective response rates as well as demonstrating safety in non-gynecologic advanced or metastatic cancers; however, data including gynecologic malignancies are limited [47,48,49].

The interactions between the TME, TSR and tissue hypoxia significantly impacts the response to radiation. The synergistic effects of combining radiation therapy, such as SBRT, with immunotherapy, have shown promising results in enhancing treatment efficacy and modulating the immune system to target cancer cells.

## 5. Immunotherapy in Gynecologic Malignancies

Chemotherapy has been a traditional systemic therapy for gynecologic malignancies with platinum-based chemotherapies, along with paclitaxel or topotecan, used as standard of care. The integration of targeted agents, such as bevacizumab, and immune checkpoint inhibitors (ICI) like pembrolizumab, have demonstrated promising results in improving overall survival and disease control in advanced or metastatic gynecologic malignancies. However, the synergistic potential of combining SBRT with ICIs remains largely unexplored in gynecologic cancers.

Bevacizumab, a vascular endothelial growth factor (VEGF) inhibitor, in addition to traditional chemotherapies, showed a significant improvement in overall survival in metastatic, persistent or recurrent cervical cancer in the Gynecologic Oncology Group (GOG) 240 phase III trial [50]. Moreover, immune checkpoint inhibitors have also shown promise in oligometastatic disease. ICIs work by blocking the interaction between the programmed death 1 (PD-1) receptor on T-cells and its ligand, programmed death receptor ligand 1 (PD-L1) on cancer cells, an interaction which suppresses the immune response. By blocking this interaction, checkpoint inhibitors allow T-cells to recognize and attack cancer cells, thereby mounting an immune response.

Pembrolizumab, a PD-1 receptor antagonist, has shown efficacy in recent trials in recurrent or metastatic endometrial cancer. The results of the KEYNOTE-028 study showed an objective response rate (ORR) of 13% in PD-L1 positive locally advanced or metastatic endometrial cancer [51]. The efficacy of pembrolizumab on PD-L1 positive tumors with high levels of microsatellite instability (MSI-H) and mismatch repair deficiency (dMMR) on a variety of primary advanced tumors types (the most common being endometrial, gastric and small intestine) was evaluated in the Phase II KEYNOTE-158 study, which showed an ORR of 30.8% with a median duration of response of 47.5 months [52]. The endometrial cohort showed an ORR of 58% and a median OS of 23.5 months [53]. The double-blind phase III KEYNOTE-826 [54] trial randomized patients with PD-L1 positive persistent, recurrent or metastatic cervical cancer to receive pembrolizumab vs. placebo in addition to standard of care chemotherapy with or without bevacizumab. Overall survival was significantly improved with the addition of pembrolizumab as compared to placebo, with a 2-year OS of 54.4% vs. 44.6% in 317 patients with PD-L1 combined positive score of 10 or more. Additionally, ipilimumab, a cytotoxic T-lymphocyte-associated antigen 4 (CTLA-4) inhibitor, as well as nivolumab, another PD-1 receptor antagonist, have shown promise in recurrent, persistent, or metastatic ovarian and cervical cancer [55,56]. Overall, the current literature regarding immunotherapy in select patients with advanced or metastatic gynecologic cancer shows moderate improvements in distant disease control without the addition of SBRT. There is one prospective clinical trial to our knowledge that evaluated SBRT with pembrolizumab in gynecologic malignancies.

The Phase II PRIMMO study [57] treated patients with persistent, recurrent or metastatic cervical or endometrial cancer with pembrolizumab, SBRT and an immunomodulatory five-drug cocktail of low-dose cyclophosphamide, aspirin, lansoprazole, vitamin D, and curcumin starting 2 weeks before concurrent radioimmunotherapy. The study consisted of 43 patients (cervical n = 18, endometrial n = 25) and the primary endpoint was the immune-related objective response rate (irORR). The results showed 11.1% and 12.0% irORR in the cervical and endometrial groups, respectively, but the median duration of response was not reached. Interestingly, patients with response had a significantly increased proportion of peripheral T-cells compared to non-responders. Although the toxicity was acceptable, with 56% grade ≥3 treatment-related toxicity, the results of this study were only modest. Our current understanding of the precise benefits of the synergy between SBRT and ICIs in gynecologic malignancies is limited. There are several ongoing clinical trials involving SBRT and ICIs, including atezolizumab in advanced or metastatic cervical cancer (NCT03614949), and tremelimumab and durvalumab in recurrent or metastatic cervical, vaginal, or vulvar cancers (NCT03452332).

In view of the current data, SBRT shows favorable local control in ROMGC, but progression outside of the radiated field remains the main site of recurrence. Current systemic therapies including platinum-based chemotherapies with paclitaxel or topotecan, with or without bevacizumab, along with immune checkpoint inhibitors such as pembrolizumab, have shown promising results. More research is needed to maximize the potential benefits of the synergistic immune-stimulating abscopal effects with SBRT. In the meantime, SBRT has consistently shown efficacy and the potential to delay systemic therapies and should be used where appropriate in select patients. As the landscape of cancer treatment evolves, a multidisciplinary approach incorporating SBRT and ICIs could potentially redefine the management of advanced or metastatic gynecologic malignancies. Leveraging the combined strength of both local control from SBRT and systemic immune response from ICIs may pave the way for more effective and personalized treatment strategies in the future.

## 6. Novel Techniques, Dose Escalation and Safety in SBRT

Magnetic-resonance-guided radiation therapy utilization is rapidly expanding, providing intra-fraction visualization, adaptive re-planning and advanced motion management, which has allowed the delivery of ultra-hypofractionated regimens. A recent Phase I clinical trial from Washington University [58], Stereotactic MRI-Guided Online Adaptive Radiation Therapy (SMART) trial, evaluated the safety and feasibility of MRI-guided adaptive SBRT for ten patients with oligometastatic ovarian cancer to a dose of 35 Gy in five fractions (BED_10_ 59.5) with optional dose escalation up to 50 Gy in five fractions (BED_10_ 100), provided organ-at-risk (OAR) constraints were met. Adaptive RT allows for real-time changes to radiation plans as MRI guidance improves visualization of tumors and surrounding OARs. The primary endpoint of this study was feasibility. The average on-table time was 69 and 54 min for plans that were adapted and not, respectively. This is comparable to other radiotherapy procedures, like brachytherapy, and all plans were able to be executed. Fifty-eight percent of fractions were adapted in this study, with the majority adapted for reversal of OAR constraint violations, which mostly occurred in the abdomen or pelvis. A single grade ≥3 toxicity was observed (duodenal ulcer). Local control at 3 months was 94% and maintained at 1 year. Median PFS was 10.9 months and systemic therapy-free survival was 11.5 months. Further research and technological advances in precision delivery of higher BEDs, while maintaining OAR constraints, could theoretically lead to increased rates and duration of local control, but the effect on overall survival from out-of-field progression is still unknown. Many studies report that SBRT is very well tolerated, with predominantly mild grade 1–2 gastrointestinal-related toxicities [13,14,17,18,22,26,27,28,29]. Grade 3–4 toxicities were noted in 2.6–10% of patients within 7/16 studies in the systematic review by Yegya-Raman et al. [12] that reported grade > 3 toxicities, and no grade 5 toxicities were reported. Acute grade 3 toxicities included duodenal ulcer, esophagitis, hemorrhagic cystitis, enterovaginal fistula and diarrhea. Late grade 3 toxicities included urethral stricture, ileus, enterocolitis, and small bowl obstructions. Grade 4 toxicities included neutropenia, hypokalemia, hyperbilirubinemia and late rectovaginal fistulas. However, Kowalchuck et al. [18] noted a single grade 5 duodenal ulcer in a patient retreated with SBRT for out-of-field recurrence to the porta hepatis (18 Gy in three fractions). Therefore, a second course of SBRT in overlapping treatment areas should be considered with caution, especially in abdominal and pelvic oligometastases. In the study by Cuccia et al. [22] 16 out of a cohort of 40 patients with endometrial and ovarian oligometastases had a repeat course of SBRT for synchronous or metachronous oligometastatic spread outside of the previously radiated field. This study showed safety in the repeat course of SBRT with no grade ≥ 2 adverse events. Repeat SBRT did not lead to a survival benefit when compared with patients who developed immediate polymetastatic spread, in contrast to lung oligometastatic disease from colorectal primary cancer with sequential oligometastases following SBRT receiving a second course of SBRT, leading to a significantly longer cancer specific survival [59].

## 7. Conclusions

SBRT is a promising and rapidly evolving treatment option in ROMGC based on multiple retrospective studies. SBRT has been shown to provide excellent local control in gynecologic oligometastases, even in chemorefractory disease. The major limitation of SBRT is progression outside of the radiated field. Given the high out-of-field recurrence rates, the safety and efficacy of the combination of SBRT with chemotherapy and immunotherapy is yet to be determined. Future prospective trials are required to elucidate the synergistic effects between these treatments. Additionally, further studies are needed to assess the potential for use of SBRT in oligometastatic gynecologic non-ovarian malignancies. While awaiting prospective trials, select patients with ROMGC should be considered for SBRT at the discretion of the radiation oncologist in agreement with multi-disciplinary discussion.
